# Is the Combination of Platelet-Rich Plasma and Hyaluronic Acid the Best Injective Treatment for Grade II-III Knee Osteoarthritis? A Prospective Study

**DOI:** 10.1155/2023/1868943

**Published:** 2023-03-10

**Authors:** Gianluca Ciapini, Matteo Simonettii, Michele Giuntoli, Giorgio Varchetta, Silvia De Franco, Edoardo Ipponi, Michelangelo Scaglione, Paolo Domenico Parchi

**Affiliations:** Department of Orthopedics and Trauma Surgery, University of Pisa, Pisa 56124, Italy

## Abstract

**Background:**

Knee osteoarthritis is a common disease with increasing incidence and prevalence in western countries. It can cause severe pain and functional limitations, thereby representing a threat for patients' quality of life and a burden for national health systems. Intra-articular injections with hyaluronic acid (HA) and platelet-rich plasma (PRP) have been used for decades in order to reduce the symptoms caused by osteoarthritis. In recent years, a combination of HA and PRP has been introduced in clinical practice with the aim to minimize the clinical presentation of osteoarthritis and potentially delay articular degeneration.

**Materials and Methods:**

Sixty cases with grade II-III knee osteoarthritis according to the Kellgren–Lawrence classification were included in a prospective study, focused on the evaluation of clinical and functional outcomes after intra-articular knee injections. Cases were randomly divided into three groups. Twenty cases (Group *A*) were injected with HA, 20 (Group *B*) had PRP, and the remaining 20 (Group *C*) received a combination of HA and PRP. Basal WOMAC score and VAS score were recorded before the treatment and repeated within 3 and 6 months after the treatment.

**Results:**

At 6-month follow-up, Group *C* (PRP + HA) was the one with the lowest WOMAC and VAS mean values. It was also the only group that reported a reduction in the two values both in the first three months and in the following three months. No major complication was recorded.

**Conclusion:**

The combination of platelet-rich plasma and hyaluronic acid can be effective in the treatment of grade II-III knee osteoarthritis in a short-to-mid-term scenario. It represents an innovative and valuable alternative to the administration of its two components alone.

## 1. Introduction

Osteoarthritis is the most common disease of joints in the adult population [1–4]. In particular, the knee is the most frequent location for osteoarthritis, as its prevalence in adults is around 6%, rising as high as 40% among the 70-to-74-year-olds [5–7]. Knee osteoarthritis thereby represents a burden for national health systems, as it can lead to serious disability caused by increasing pain and progressive articular stiffness that may limit patients' autonomy and thereby have a negative impact on their quality of life [8–12]. The therapeutic goal for those cases that suffer from mild-to-moderate knee osteoarthritis is to alleviate the signs and symptoms of the disease and, if possible, to slow its progression. In this scenario, intra-articular injections with anti-inflammatory or viscosupplementation drugs represent a therapeutic option capable of reducing patients' pain and restore part of their previous functionality, as testified by several meta-analyses [13, 14] and randomized controlled trials [15, 16].

Discovered in 1934 by Karl Meyer and John Palmer, the hyaluronic acid (HA) has been used since the 1970s to treat articular pain due to mild and moderate knee osteoarthritis. For almost half a century, HA has been one of the most used injective drugs for the treatment of osteoarthritis considering both its physical-chemical properties and its interaction with cartilages' cells and extracellular matrixes (ECMs) [17]. In the last decades, the continuous search for the discovery of new and effective injective treatments has driven the interest in regenerative biologic therapies [18]. Autologous platelet-rich plasma (PRP) has been introduced as an alternative to HA for cases suffering from moderate knee osteoarthritis, leading to encouraging results in terms of both pain relief and functional recovery [19, 20]. A further innovation of the two approaches is represented by the administration of PRP alongside HA, introduced in recent years and performed with the theoretical aim to combine the effects of these two drugs in order to maximize and extend their efficacy. To this date, only a limited number of studies have evaluated the effectiveness of this combination despite some encouraging preliminary results [21–25]. The aim of our study is to evaluate the effectiveness of the association of PRP and HA, comparing it with its two components administered alone. The effectiveness of injective treatments was assessed through the months that followed their administration in a single institution on similar cohorts of patients suffering from comparable grades of knee osteoarthritis (grade II-III according to the Kellgren–Lawrence classification). Providing the best possible standardization between the three groups (HA, PRP, and PRP + HA), we investigated whether the combination of PRP and HA could be more effective than PRP or HA alone in terms of functional recovery and pain control on short-term and mid-term scenarios.

## 2. Materials and Methods

This single-center double-blinded prospective study was approved by our Ethical Committee (CEAVNO CE150034-835/2015) and performed in accordance with the ethical standards laid down in the 1964 Declaration of Helsinki and its later amendments. All patients gave their written consent. The study was conduced between January 2018 and January 2020.

Inclusion criteria for the inclusion in our research were a radiographic evidence of knee osteoarthritis (grade II-III according to the Kellgren–Lawrence classification), pain or functional limitations in patients' activity of daily living before the treatment, being between 30 and 80 years of age, and the absence of clinical or imaging signs of articular instability. Exclusion criteria were known hypersensitivity to hyaluronic acid, pregnancy and lactation, a BMI higher than 40, chronic administration of anticoagulant drugs or a history of coagulopathies, neoplastic lesions, and knee or kidney failures.

Sixty eligible cases were included in our study. Gender, age, and body mass index (BMI) of each patient were recorded at the moment of their inclusion in our study. The clinical picture of each case was analyzed before the treatment using the Western Ontario McMaster Universities Osteoarthritis Index (WOMAC) to evaluate patients' overall clinical condition and the visual analogue scale (VAS) to assess their pain in particular.

Patients were randomly divided into three groups, with 20 subjects (10 males and 10 females) for each group. Group *A* was treated with intra-articular injections of hyaluronic acid (HA) (ArthroVisc; Regen Lab, Le Mont-sur-Lausanne, Switzerland), and Group *B* was treated with intra-articular autologous platelet-rich plasma (PRP) (RegenKit-BCT-1; Regen Lab, Le Mont-sur-Lausanne, Switzerland), whereas Group C received a combination of platelet-rich plasma and hyaluronic acid (Cellular Matrix A-CP-HA kit; Regen Lab, Le Mont-sur-Lausanne, Switzerland).

The drug was administered in our clinic on a monthly basis, for a total of three intra-articular injections through two months. The injections were performed in sterile conditions. Every eventual adverse reaction to the drugs was recorded and reported. Further outpatient visits were performed three and six months after the last injection, evaluating each patients' clinical outcomes in terms of VAS and WOMAC scores. These values, compared to the ones obtained before the treatment, were used to assess the effectiveness of the combination of PRP and HA compared to the two components administered alone.

### 2.1. Materials

The hyaluronic acid used in our study was ArthroVisc (Regen Lab, Le Mont-sur-Lausanne, Switzerland). The product consists of a 2 ml syringe containing 40 mg of fermented hyaluronic acid with a molecular weight of 1550 kDa.

The administration of PRP alone was performed using the RegenKit-BCT-1 (Regen Lab, Le Mont-sur-Lausanne, Switzerland). For each patient, 8 ml of peripheral circulating blood was collected in a proper tube and centrifuged at 1500g for five minutes, obtaining 4 ml of platelet-rich plasma. The PRP was then drawn into a sterile syringe and injected in the patient's knee.

The injections of PRP alongside HA were performed using the Cellular Matrix A-CP-HA kit (Regen Lab, Le Mont-sur-Lausanne, Switzerland). The kit was provided with tubes that allowed to mix 2 ml/40 mg of hyaluronic acid (1550 kDa) with 4 ml of platelet-rich plasma obtained with the aforementioned process. At the end of the preparation phase, a sterile syringe containing 4 ml of PRP and 2 ml of HA was injected in the target knee.

All the three kits used in our study were produced by the same company. The chemical properties of the hyaluronic acid injected alone were the same as the one that was combined with PRP. The tubes and the other devices of the kits used to prepare PRP and PRP + HA were comparable, and both kits were used with the same centrifugation system.

### 2.2. Statistics

Statistical analysis was performed using Stata SE 13 (StataCorp LLC). Statistical significance was set at 0.05 for all endpoints. Descriptive analyses were performed for each value of VAS and WOMAC scores. *T*-student tests were used to assess eventual differences between the results obtained by those who received PRP + HA and the ones obtained by other cohorts.

## 3. Results

The mean age of our 60 cases was 60.2 years (39–80), while the mean BMI was 25.5 (19–36). The average starting VAS and WOMAC scores of the whole population were 5.6 (1–9) and 37.2 (5–85), respectively. None of our cases developed adverse symptoms or signs attributable to the received treatments.

**Figure 1:**
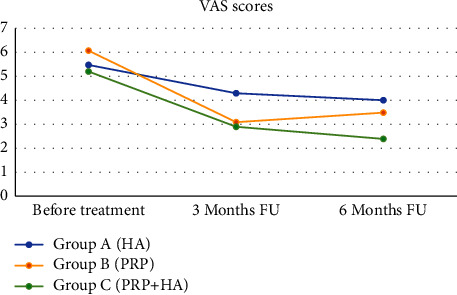
The evolution of the mean visual analogue scale (VAS) scores in the three groups. HA: hyaluronic acid, PRP: platelet-rich plasma, and FU: follow-up.

**Figure 2:**
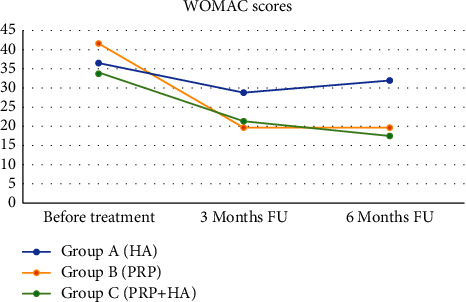
The evolution of the mean Western Ontario McMaster Universities Osteoarthritis Index (WOMAC) scores in the three groups. HA: hyaluronic acid, PRP: platelet-rich plasma, and FU: follow-up.

Cases belonging to Group *A* (HA) had a mean basal VAS score of 5.5, a value that decreased to 4.3 after three months and 4.0 after six months (Figure 1). Their mean basal WOMAC score was 36.4 at base time, 28.8 after three months, and 31.8 after six months (Figure 2). Cases of Group *B* (PRP) had a mean VAS score of 6.1 before injections, 3.1 after three months, and 3.5 after six months (Figure 1). Their WOMAC score was 41.5 at the beginning of our study and 19.6 after three months, remaining steady three months later (Figure 2).

Cases in Group *C* (PRP + HA) saw a distinct reduction of their mean VAS score through our study, moving from a starting value of 5.2 to 2.9 after three months to 2.4 after six months (Figure 1). The mean WOMAC score decreased from 33.7 to 21.4 at three-month follow-up and later to 17.5 at final six-month follow-up (Figure 2).

The aforementioned results are summarized in Table 1.

The mean differential VAS score between the starting value and the one recorded six months after the end of the treatment was 1.6 in Group *A*, 2.6 in Group *B*, and 2.8 in Group C (Figure 1).

One-tailed *t*-student tests on VAS scores taken six months after treatments testified a significantly better pain relief in Group *C* compared to both Group *A* and Group *B* (*p*=0.005, *p*=0.043). These outcomes suggest that, at a mid-term follow-up, a combination of PRP and HA provides better pain control compared to PRP and, in particular, HA alone.

At three-month follow-up, cases of Group *A* had higher mean WOMAC scores (36.4) compared to the ones of Group *B* (19.6) and Group *C* (21.4). These data highlighted a first difference between cases treated with HA alone and the rest of our cohort, although they were not statistically significant to that point due to the limited size of our population (*p*=0.076). Three months later, the mean score of cases treated with HA (Group *A*) was 31.8, the one of those who received PRP (Group *B*) was 19.6, and the one of those who received a combination of the two (Group *C*) was 17.5. Therefore, at this final endpoint, those who were injected with PRP and HA had the best functional outcomes according to the WOMAC scoring system. Furthermore, Group *C* was the only one whose mean value continued to decrease after the first three months that followed the treatment, suggesting a prolonged effectiveness of the combined approach. Although the difference between Group *B* and Group *C* was not sufficient to be considered statistically significant according to our endpoints (*p* > 0.050), the treatment with PRP (Groups *B* and *C* together) resulted to be significantly better compared to HA alone in terms of clinical outcomes within six months from the end of the treatment.

## 4. Discussion

The combination of HA and PRP represents one of the most promising injective approaches introduced in recent years for moderate knee osteoarthritis, as it could theoretically dovetail the positive characteristics of both of its components. The hyaluronic acid, a natural component of the synovial fluid, can increase joint viscosity and lubrication but also reduce articular inflammation [21]. However, although literature provides evidence that intra-articular injections with HA can ease the symptoms associated with mild-to-moderate osteoarthritis, there is no evidence that the drug could anyhow halt the progression of the disease and these effects tend to decrease months after the treatment [25]. Our results are in line with the ones reported in the literature, as our cases treated with HA injections experienced good results at 3-month control in terms of both pain relief and restoration of knee functionality. However, the same patients did not report further significant improvements in the next 3 months. In fact, 6 months after the treatment with HA, patients did not experience any significant further pain reduction (according to the visual analogue scale) and they even reported a slight worsening of their knee functionality (according to the WOMAC score).

Platelet-rich plasma, for its part, is another suitable option for today's intra-articular injection treatment in mild-to-moderate knee osteoarthritis. Despite the limited standardization of its production and formulation that limit its introduction in most guidelines, PRP has been shown to reduce pain and inflammation associated with knee osteoarthritis. The literature provides evidence that intra-articular administrations of PRP may influence tissue regulation due to the high level of platelet-derived growth factors [8, 17, 19–21]. Our population also confirms PRP's short-term effectiveness. Comparing the base-time evaluations and the ones at three-month follow-up of all our cohorts, the one treated with PRP had the greatest increase in both VAS and WOMAC scores, thereby suggesting the short-term effectiveness of PRP. Although the WOMAC of those patients remained steady in the following three months and VAS scores even worsened at six-month follow-up, both these scores were higher and had greater improvements from the basal values in comparison with the ones of those cases who received HA injections.

In recent years, the dualism between PRP and HA has been partially overcome, as the injection of a combination of the two materials has been proposed and introduced in clinical practice. However, only a limited number of prospective studies have been published so far on this topic [22–27]. In 2016, Saturveithan et al. [22] compared the effects of a cycle of PRP + HA with the ones of HA alone in cases suffering from grade III-IV knee osteoarthritis. The authors reported that those who received PRP alongside HA had much better outcomes in terms of both knee functionality and pain relief within six months after the end of their treatment. Three years later, in 2019, Papalia et al. [23] published a study with a similar design held on 60 cases suffering from mild-to-moderate knee osteoarthritis (grade II and III according to the Kellgren–Lawrence classification). The authors demonstrated better functional outcomes within 6 months from the treatment for those who received PRP + HA rather than HA alone.

Our study, performed on cases suffering from the same stages of osteoarthritis (grade II and III according to the Kellgren–Lawrence classification), did not comprehend only two cohorts that received HA and PRP + HA but also included a third subpopulation that received only PRP, allowing a comparison between the three treatments in a mid-term scenario. In our population, at six-month follow-up, cases treated with intra-articular injections of PRP + HA had significantly lower VAS scores compared to the other cohorts and the absolute lowest WOMAC scores. Furthermore, cases that received the combination of the two treatments were the only ones that reported a remarkable advancement of their functional recovery and pain relief between not only in the first three months but also between the three and six months after the treatment.

These results are in line with the ones already reported in the literature by Lana et al. and Yu et al. [24, 25], who carried out studies with comparable designs. However, our study differs from the first one in terms of knee osteoarthritis grade. Lana et al. evaluated cases with grade I to III according to the Kellgren–Lawrence classification, whereas our choice was to exclude cases with incipient knee osteoarthritis, rather focusing on those who were already suffering from mild-to-moderate disease (grade II-III), since they represent the vast majority of those who get the indication for intra-articular injections in our institution. The paper by Yu et al., for its part, did not specify any radiographic grade of knee osteoarthritis among its inclusion criteria. This does not allow a proper comparison between our and their population, despite the convergence of our results. Further randomized controlled studies are going to provide further evidence on the topic in the near future [26].

We are aware that our study is not free of limitations. Although the size of our population (made of 60 cases divided into 3 groups of 20 cases each) is globally comparable to the ones of previous studies on the topic, the lack of greater cohorts represented a restriction for the statistical significance of our outcomes. Another limit lies in the duration of our follow-up. Since our endpoint was set to 6 months after the end of injective treatments, we could not extend our horizon any further in order to assess the effectiveness of PRP + HA in a long time period.

Altogether, our prospective study testifies the effectiveness of PRP + HA for cases suffering from grade II and III knee osteoarthritis, as patients' overall outcomes in terms of pain control and functionality resulted to be better than comparable control groups treated with HA and PRP alone in similar conditions. Our experience suggests that a larger use of combined PRP and HA could lead to significative benefits for patients with mild-to-moderate knee osteoarthritis. Better pain control and functional performances would translate into a quality of life but also in reduced healthcare spendings. Faced with the reasonable costs of some injections, the treatment with PRP and HA could allow the national health systems to reduce their expenses in terms of painkillers and outpatient visits in the months that follow the treatment.

## 5. Conclusions

Our study suggests that the combination of platelet-rich plasma and hyaluronic acid represents an innovative and valuable alternative to the administration of its two components alone. Being capable of reducing both pain and functional impairment within the 6 months that follow its administration, this treatment represents a promising frontier for the nonsurgical approach for patients who suffer from mild-to-moderate knee osteoarthritis.

## Figures and Tables

**Table 1:** Summary of the mean visual analogue scale (VAS) and Western Ontario McMaster Universities Osteoarthritis Index (WOMAC) values in our population, sorted per group.

Time	VAS score (0–10)			WOMAC score (0–96)		
	*t *0	3 M	6 M	*t *0	3 M	6 M
Group *A* (HA)	5.5 (1–9)	4.3 (1–9)	4.0 (0–8)	36.4 (7–77)	28.8 (2–84)	31.8 (1–80)
Group *B* (PRP)	6.1 (2–9)	3.1 (0–6)	3.5 (0–6)	41.5 (5–85)	19.6 (0–42)	19.6 (0–45)
Group *C* (PRP + HA)	5.2 (1–8)	2.9 (0–6)	2.4 (0–5)	33.7 (7–73)	21.4 (5–45)	17.5 (4–45)

HA: hyaluronic acid; PRP: platelet-rich plasma. *t*0 = base time; 3 M = 3 months; 6 M = 6 months.

## Data Availability

The data used to support the findings of this study are available from the corresponding author upon reasonable request.
